# Stage distinctive communication networks of the online breast cancer community

**DOI:** 10.1038/s41598-023-28892-7

**Published:** 2023-01-31

**Authors:** Wonkwang Jo, Sou Hyun Jang, Eun Kyong Shin

**Affiliations:** 1 Department of Public Health Sciences, Graduate School of Public Health, Seoul National University, Seoul, South Korea; 2 Institute of Health and Environment, Seoul National University, Seoul, South Korea; 3 Department of Sociology, Korea University, 145 Anam-Ro, Seongbuk-Gu, Seoul, 02841 South Korea

**Keywords:** Breast cancer, Health care

## Abstract

In this study, we reveal the distinctive communication network structures and contents of online breast cancer community posts in accordance with different cancer stages. Using data collected from community.breastcancer.org, a major online breast cancer community (28,139 original posts and 663,748 replies), we traced the communication network structures and contents of replies associated with its severity. By combining network and quantitative content analyses, we deciphered the functions and utilities of health-related online communication. We found an inverse relationship between offline epidemiological prevalence and online communication activation. Despite the relatively small percentage of breast cancer patients, it was found that the more severe the condition of breast cancer, the more active online communication was. We further found that as pathological severity advances, communication networks move from informational exchange to emotional support. The capture of online social networks based on the cancer stage can help unpack the distinctive communication patterns found across different cancer severities. Our results provide insights into a possible online communication intervention design tailored to symptom severity.

## Introduction

With the development of the Internet, people can now join and actively interact with members of various groups on communities in social media, such as education, sports, and work groups on Facebook^1^. Online health communities (OHCs) have also become crucial places for patients and their significant others, such as family members and friends, to communicate and interact with each other. OHCs can not only fill the gap in the offline care system^2,3^ but can also serve as a critical nexus to obtain information and provide support to patients with breast cancer. OHC platforms are particularly important in disadvantaged situations where there is limited access to medical resources and information^4^.

OHCs are highly associated with patients with breast cancer owing to their high incidence and survival rates. Breast cancer is the most common cancer in the U.S.^5^ and is ranked third in mortality, after lung and pancreatic cancers^6^. Although approximately 271,000 individuals were diagnosed with breast cancer in 2019, the survival rate (90%) for breast cancer was relatively higher than for lung and pancreatic cancers (19% and 9%, respectively)^7^. The increasing incidence and survival rates imply that many individuals diagnosed with breast cancer remain patients for a long time. Prolonged patient and survivor status has made breast cancer one of the most actively utilized online health community topics^2,8^, and has provided patients with ample opportunities to build digital networks.

Online breast cancer communities have been widely studied across multiple disciplines^9–11^. They exist in a wide variety of forms in different countries, providing patients and their friends, families, relatives, and other healthcare consumers, opportunities to share information about their health conditions and wellbeing^2,8^. Considering that breast cancer is a disease in which patient health behavior makes a critical difference in prognosis^12,13^, it is natural for scholars to study the influence and function of OHCs.

Most existing studies have involved a qualitative content analysis or surveys of OHCs^3,9,10^. Previous research focusing on OHCs (not users) has mainly dealt with the communication *contents* produced in communities. For example, Chen applied clustering algorithms to the online discussion board content of three diseases to determine the overall content. Similarly, Gooden and Winefield divided the content of posts written by the participants into two categories^9,10^.

The *communication network structure* of OHCs is as crucial as their *content*. First, the communication network structure can affect the behavior of users, as has been highlighted by many scholars. For example, Centola proposed that the clustered network structure of communication in the online community is beneficial in promoting and disseminating health behaviors when compared to the random network structure^14^ because clustered networks contain many redundant links, which leads to repeated stimuli, thus inducing the required behaviour^15^. Second, we can infer the characteristics of user activities from the communication network structure because they reflect the features of the activities. For example, communities with a primary focus on information delivery and emotional exchange have different communication network structures. Therefore, by analyzing the communication network structure of an online community, we can understand the purpose and function of communication within the community.

In this study, we analyzed the online breast cancer community’s content and communication network structure. By studying both factors, we expect to understand the impact of the online community and the characteristics of the activities occurring within it in more detail than in previous studies. To delve into the structure and content of the communication network, we focused on replying activities. In an online patient community, the primary way users interact and communicate is by responding to other people’s posts. Through this process, users form a post-reply network. An analysis of its structural characteristics and an uncovering of how content is communicated through replies constitute an essential way of understanding the utilities of online communities. Additionally, the content of the replying posts reveals the communication content among users. Therefore, in this study, we focused on the replying activity’s content and communication network structure.

We examined the differences in communication content and communication network structure during different stages of cancer. Patients’ needs and concerns are diverse depending on their disease stage, and they call for differential support (informational, social, and emotional) at each stage^16,17^. Approximately two-thirds (64%) of breast cancer patients had local stage (or stage 1) breast cancer, slightly more than a quarter (27%) had a regional stage, and 6% had distant stage^18^. Patients with breast cancer in different stages exhibit considerable variance in prognosis and are exposed to different pressures in implementing the required behavioral changes for recovery^19^. The information needs^20^ and the need for support (informational, social, and emotional), are different at each stage. For example, breast cancer patients at late stages are more likely to be socially isolated^21^; thus, they are more likely to seek online social support than breast cancer patients at early stages^22^. Furthermore, both breast cancer patients and their families are likely to be stressed and burdened in later stages of illness^23,24^. However, the scarcity of empirical evidence on how the differential needs pertinent to each cancer stage influence OHC activities limits the effectiveness of care design for cancer survivors.

A stage-specific examination of the distinctive patterns in the contents and structures of communication pertinent to different stages allows us to unveil the digital environment more accurately. In doing so, we hope to contribute to the understanding of the association between online communication and disease severity and develop more efficient and effective intervention strategies. Thus, we measure the content of the replies and the communication network structure of the replying activities in the breast cancer online community and analyze how they vary depending on the severity of the condition. Through this, we reveal the detailed characteristics of the activities occurring in the online community and infer their effects and policy implications.

## Materials and methods

### Data

In this study, we investigated the breast cancer-related communication network structure using multiple forums of the nationwide online breast cancer community (i.e., community.breastcancer.org). As of September 20, 2021, community.breastcancer.org was the largest online breast cancer community, with more than 223,833 members, 163,223 topics, and 83 forums. In this community, a forum refers to a separate bulletin board focusing on a specific subject (e.g., mastectomy, reconstruction surgery); a topic refers to a bundle of original posts and replying posts (e.g., a topic = an original post + 20 replying posts to the original post). By posting and replying in various forums, members become acquainted with each other, comfort each other, and exchange necessary information.

Our study aimed to analyze the differences in the contents and network structure of online communication in online breast cancer community forums according to the stages of breast cancer. Of the 83 forums, we focused on the four forums themed according to the different cancer stages on community.breastcancer.org. The forums’ names were as follows: ‘Stage I Breast Cancer,’ ‘Stage 2 Breast Cancer,’ ‘Stage 3 Breast Cancer,’ and ‘Stage IV/Metastatic Breast Cancer ONLY.’ Although those who do not fall under the relevant stages can write posts on each of the forums, we assumed that the forums’ replying posts show the contents and structure of online communication as per the cancer stage because it is presumed that the patients belonging to the stage or people related to them (family members or friends) are the ones who mainly use the forum. Identification of the differences or similarities in the communication networks or contents of the four forums contributes to analyzing the effect of a cancer stage on communication.

We created a simple scraping program to collect all available posts from the four forums, accessed August 2021. We extracted two pieces of information from the collected posts. The first was the text content in posts that replied to the original posts in the forums. The second was the user communication network, estimated using the replying activity. As such, we created a user communication network based on the following principles.

When someone writes a replying post on the original post, the ID of the person who wrote the reply is connected to that of the person who wrote the original post. If A writes a post and B, C, and D reply to the post, we assume that there is a communication network in the manner of B → A, C → A, and D → A. We defined the link weight between A and B (A → B) as the number of replying posts of A to B. Further, we developed separate communication networks for each forum. In short, user IDs were nodes, and the link is assumed based on the replying activity.

We extracted the words that appeared in the replying posts using R packages called tidytext and tokenizer^25,26^. We only used words containing at least one alphabet letter as data. We applied a stemming process to the words using the Snowball C package^27^ to modify multiple words from the same stem into the same form (e.g., family, families—> famili).

### Analyzing methods

We used three methods. (1) We identified the most frequently appearing words for each forum’s replying posts, (2) measured the structural characteristics of the communication network using various indicators, and (3) tested the significance of the differences in indicators depending on the stage. First, the most straightforward way to analyze the content of comments was to pay attention to the words used. We analyzed the characteristics of the communication content of each forum by checking the words that appeared most frequently in each forum’s replying posts.

Second, we measured the characteristics of the communication networks created for each forum using various network indicators. The network indicators measured included the global clustering coefficient, average local clustering coefficient, reciprocity, and mean distance. A brief description and application background of each measure are as follows.

The global clustering coefficient indicates the number of closed triangular structures in an entire network^28,29^. It is defined as the following:

$$Global clustering coefficient= \frac{\left(number of triangles\right)X6}{(number of paths of length two)}$$

The triad can be interpreted as the smallest structure with the character of a society because the triad embeds dyadic relations in a multilateral relation^30^. In social networks, which type of triadic relationship is prevalent is an important question. Among the various types, closed triangles imply the transitivity of relation and interaction, which means that if A interacts with B, and B interacts with C, then A also interacts with C. The fraction of this pattern in a network, which the global clustering coefficient measures, indicates the number of nodes that tend to make tightly knit groups. We assume that a large value of clustering coefficients indicates the existence of cohesive groups because it means that users are more likely to interact with their neighbors’ neighbors if we define a user’s neighbors as other users who interact with the user.

Unlike the global clustering coefficient, the local clustering coefficient was measured for each node. This is the ratio between the number of connected pairs of neighbors of a node and the number of pairs of neighbors of a node^28^.

$$Local clustering coefficient of node i= \frac{(number of connected pairs of neighbors of i)}{(number of pairs of neighbors of i)}$$

For example, if a node is connected to four nodes, the local clustering coefficient measures the number of links existing among the four nodes and divides the number by six, which is the maximum number of links among the four neighbors. This can be considered an indicator of the cliquishness of friendship circles at the node level^31^. The overall average of this indicator can estimate the tendency of clusters in the entire network at a different angle from the global clustering coefficient.

Reciprocity measures how many reciprocal connections exist in a directed network^28^. A reciprocal connection means that if there is a link A → B, then a link B → A also exists. If reciprocity is high, the network has more reciprocated links, which could mean that network members impose expectations and obligations on each other.

The mean distance is the average distance of a pair of nodes existing in the network^29^. We calculated the distance by considering the direction and averaged the distances. In this process, we do not consider disconnected node pairs. We only averaged the distances between the connected node pairs. We also did not consider weight. The shorter the average distance, the closer the forum users were, and the more active their communication was with each other.

We measured the values of the four indicators and analyzed how they differed depending on the stage. Furthermore, we conducted an additional significance test through network randomization to determine whether the difference in the communication network at each stage was accidental or significant. We performed network randomization through link rewiring with the degree sequence of each communication network fixed^32–35^. The number of link rewiring attempts was ten times the number of links of the observed network. We repeated this process 1,000 times per network; therefore, we created 1,000 simulated networks for each communication network. These are random networks of the same size and degree distribution as that of each communication network. By measuring the four indicators mentioned above in these networks, we can obtain a sort of random distribution for the four indicators in each communication network. We can also obtain the distribution of the differences in the four indicators between different communication networks.

We tested the significance of the observed differences in the indicators based on the distributions of the simulated networks. Similar to a conventional two-sided significance test, we determined significance based on how many larger differences were found than the observed differences from the sets of simulated networks. As previously mentioned, we created 1,000 simulated networks per stage to have 1,000 values for each indicator per stage. With these values, we can calculate 1,000 differences in each indicator between stages, which can be interpreted as a random distribution of the difference in each indicator. If we found fewer than 25 cases with more extreme differences than the observed difference in an indicator, we determined that the observed difference in the indicator was significant at a level of 0.05. If we found fewer than five cases with more extreme differences than the observed difference, we determined that the observed difference in the indicator was significant at the 0.01 level. If we did not find a case with a more extreme difference than the observed difference, we determined that the observed difference was significant at the 0.001 level.

Finally, to express the network structures of the forums more intuitively, simple network graphs were created with the most significant links for each forum. After identifying the top 200 links based on the link weights of each forum’s replying network, we created four subnetworks with these links in each forum. The largest component in each subnetwork was visualized. The network layout for visualization was that of Fruchterman and Reingold^36^.

We utilized the R ^37^ and R packages for all analyses. The list of packages is as follows: httr, rvest, tidyverse, tidytext, slam, SnowballC, igraph, ggraph, and cowplot^25,27,38–44^. All methods were performed in accordance with the Declaration of Helsinki and the relevant guidelines.

### Ethical statement

This study was reviewed and approved by the Sungkyunkwan University Institutional Review Board (IRB: #NON2021-001).

## Results

The total numbers of original posts and replies recorded in each forum (‘Stage I Breast Cancer,’ ‘Stage II Breast Cancer,’ ‘Stage III Breast Cancer,’ and ‘Stage IV/Metastatic Breast Cancer ONLY’) are presented in Table 1. In terms of the number of original posts, online communication was the most active in the fourth stage forum, followed by that in the third, first, and second stage forums.

**Table 1:** Number of posts in each forum.

Forum	Number of original posts (topics)	Number of replying posts	Number of replying posts per original post (mean)
Stage I Breast Cancer	1229	36,150	29.4
Stage II Breast Cancer	302	10,267	34.0
Stage III Breast Cancer	3782	66,455	18.0
Stage IV/Metastatic Breast Cancer ONLY	22,826	550,876	24.1

We found an inverse relationship between offline prevalence and online activation; the more severe the condition of breast cancer, the more active the use of forums. In the U.S., most patients are diagnosed with breast cancer at an early stage, and more than 75% are diagnosed at the local stage^45^. According to clinical breast cancer statistics, the proportion of patients with stage four terminal disease (distant stage) is approximately 6%^7^. Nevertheless, 81.1% of the original posts were associated with stage four breast cancer, and 13.4% were associated with stage three of the disease.

We measured key network indices to detect differences between communication structures. Details of the operationalization are provided in the Methods section. The structural statistics of the communication networks are listed in Table 2. We identified four distinctive network patterns across the different cancer stages. The first is the number of nodes and links. As shown by the total number of posts, the number of nodes and links in the fourth stage forum was the highest. We found that more active communication in the forum was related to the most advanced cancer stage. The average number of links per node also increases by stage, implying that in the later stages of forums, users interact with various users. Second, global and local clustering coefficients were higher in forums related to the later stages. This indicates that the communication networks of the later-stage forums show more clusters, and there are many redundant links. Third, reciprocity was higher among later-stage forums. That is, in the later stages of forums, users tend to write a replying post reciprocally. Finally, the average distance among nodes tends to be lower in the later-stage forums than in the earlier-stage forums.

**Table 2:** Descriptive statistics of the communication networks of each forum.

Forum	Stage I Breast Cancer	Stage II Breast Cancer	Stage III Breast Cancer	Stage IV/Metastatic Breast Cancer ONLY
Number of nodes	4565	1568	5020	12,284
Number of links	10,585	2636	26,620	147,513
Global clustering coefficient	0.0100	0.0103	0.1225	0.1645
Reciprocity	0.0079	0.0046	0.1243	0.2193
Average local clustering coefficient	0.0569	0.0522	0.2227	0.3325
Average distance between nodes	5.1112	5.0259	3.1526	3.0610

**Figure 1:**
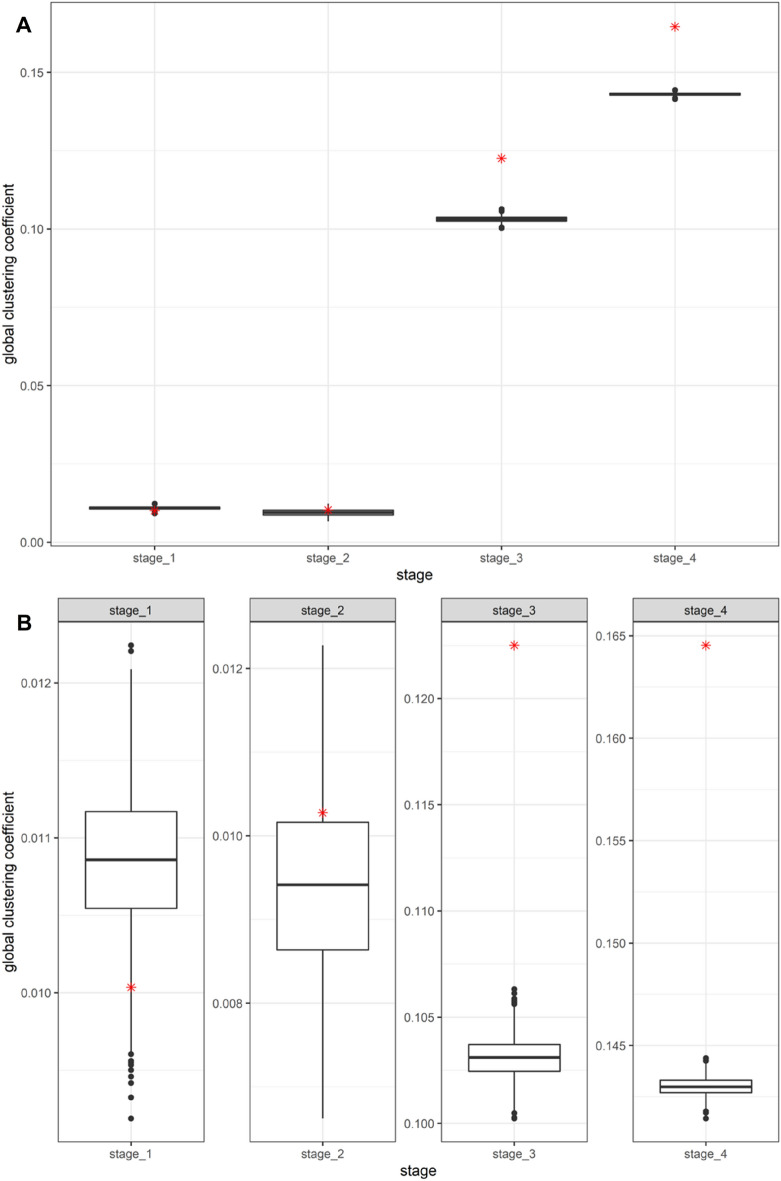
The distributions of global clustering coefficients and observed values.

We attempted a significance test based on network randomization to determine whether the observed differences in the indicators were accidental or statistically significant. As mentioned in the Method section, we obtained the distribution of the four network indicators through network rewiring with a fixed degree sequence. We also analyzed the distance between the distributions and observed values. Figure 1 shows the results for the global clustering coefficient. The boxplots are the distributions of the global clustering coefficients obtained from the simulated networks, and the red dots are the observed values. Panel A expresses the distributions of the global clustering coefficients from four forums at once, and panel B zooms in each distribution and the observed value by varying the range of the y-axis. We found that the global clustering coefficients from the communication networks of stages 3 and 4 were exceptionally high, out of the distributions from rewired networks. The results for the other three indicators are shown in Supplementary figures S2, S3, and S4.

**Figure 2:**
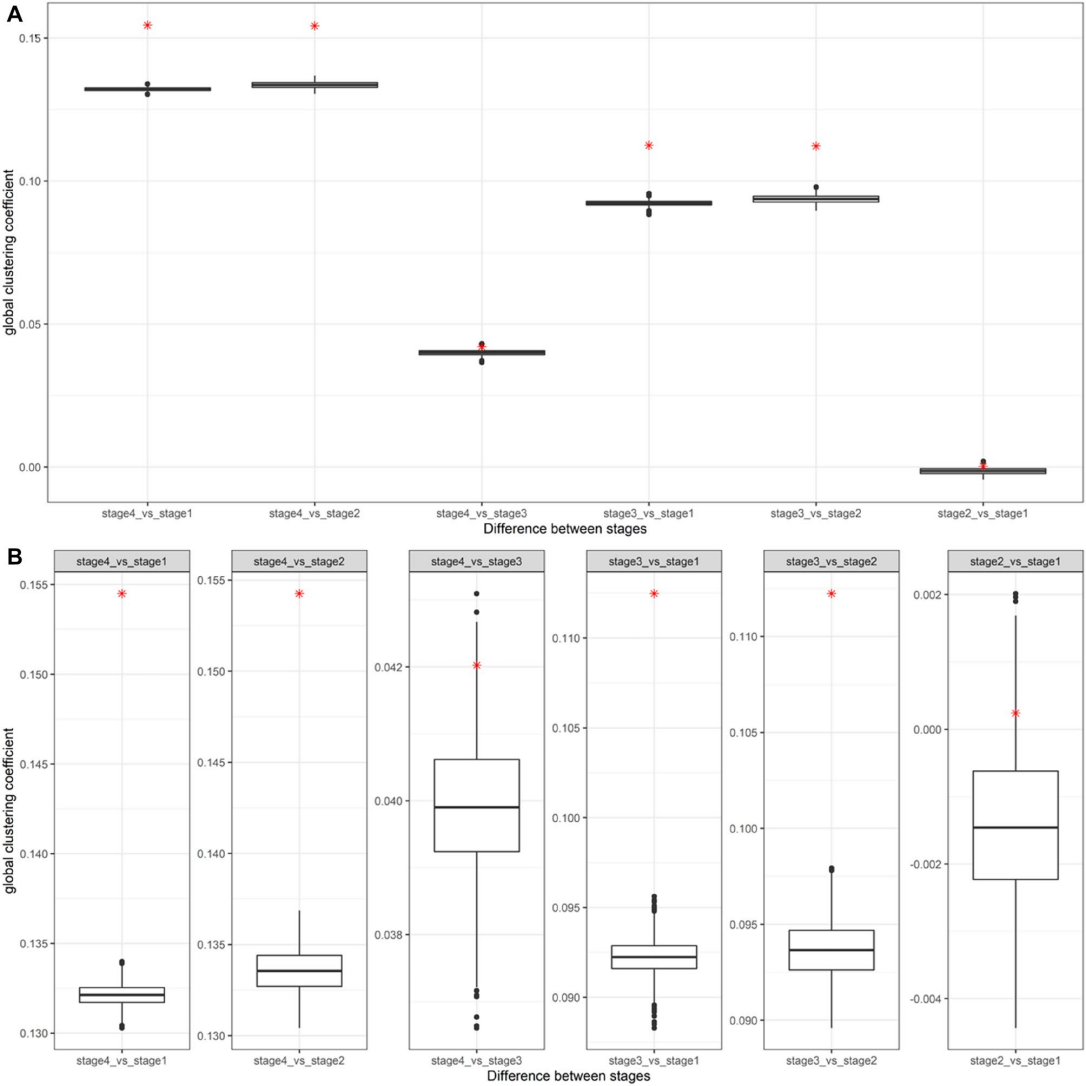
The distributions of differences in global clustering coefficients between stages and observed values of difference.

We attempted significant tests on the differences of four network indicators (global clustering coefficient, local clustering coefficient, reciprocity, and distance) between forums. As there are four forums and four communication networks, we obtained six pairs (2–1, 3–1, 3–2, 4–1, 4–2, 4–3) for testing. We produced distributions of four indicator differences in these six pairs and tested the significance of the currently observed indicator differences based on them. Figure 2 presents the results of the global clustering coefficients.

We can find the significance of the differences between the late and early stages. The observed differences between Stages 4 and 1, 4 and 2, 3 and 1, and 3 and 2 are larger than all the simulated differences. This means that the difference in the clustering coefficients between the late and early stages was significant. The other indicator figures can be found in the supplementary information (Supplementary figures S6, S7, and S8).

We summarize the significance test results for the differences in the four indicators in Table 3. Except for the mean distance, we found significant differences between the early and late stages of the three indicators. Both clustering coefficients and reciprocity were significantly higher in the third and fourth stage networks than in the first and second stages. Furthermore, the fourth stage network showed significantly different values for all four indicators from the third stage network. Conversely, there were no significant differences in any indicators between the first and second stages.

**Table 3:** Descriptive statistics of the communication networks of each forum.

Difference pair	Average_local_CC	Cluster_coef	Mean_distance	Reciprocity
Stage2 vs. Stage1	−0.00473	0.00024	−0.085336	−0.003383
Stage3 vs. Stage1	0.16576 ***	0.11247 ***	−1.958664	0.116332 ***
Stage3 vs. Stage2	0.17049 ***	0.112229 ***	−1.873328	0.119715 ***
Stage4 vs. Stage1	0.275611 ***	0.154494 ***	−2.05021	0.211408 ***
Stage4 vs. Stage2	0.280341 ***	0.154254 ***	−1.964874	0.214791 ***
Stage4 vs. Stage3	0.109851 ***	0.042025 *	−0.091546 ***	0.095076 ***

“Stage A vs. Stage B” indicates the value of Stage A minus the value of Stage B; ***p < 0.001, **p < 0.01, *p < 0.05.

**Figure 3:**
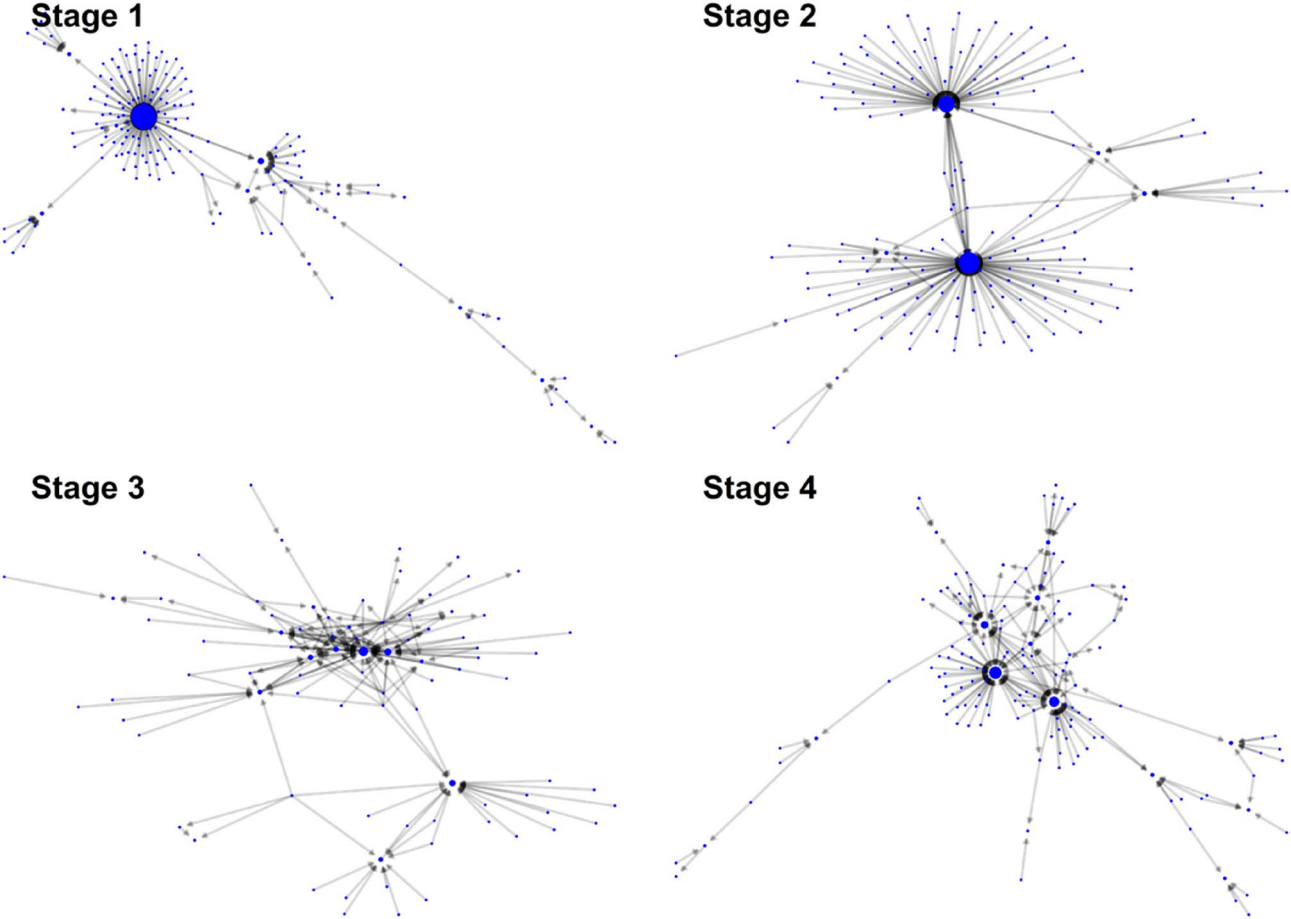
Communication networks by the stages of cancer.

Visual representations of the networks are shown in Fig. 3. Each network grasps the baseline connecting patterns in each forum. Given large network sizes (thousands of nodes and more links), the entire network image of each forum is indecipherable. Therefore, we visualized the simplified networks of each forum. Simplified networks are composed of links with high weights. Detailed information on how to create simplified networks is presented in the Methods section. The node’s size in the graphs is proportional to the in-degree of a node. That is, a node receiving more replying posts is larger in the graphs.

In the case of communication networks observed in the first and second stage forums, many people send replying posts to a small number of important nodes; however, the people who reply do not tend to write replies to other commenters. Although central nodes were observed in the third and fourth stage forums, their relative importance decreased, and communication among the repliers was notable in the case of more triangular interactions.

To further assess whether these distinctive communication patterns were related to the content, we calculated the word frequencies in the reply posts in each forum. Table 4 shows the 30 most frequently used words in the reply posts for each forum. The endings of several words are changed because of the stemming process, which modifies multiple words from the same stem into the same form (e.g., family, families—> famili).

**Table 4:** Top 30 words based on the frequency of use in each forum.

Stage I Breast Cancer		Stage II Breast Cancer		Stage III Breast Cancer		Stage IV/Metastatic Breast Cancer ONLY	
Word	n	Word	n	Word	n	Word	n
chemo	13,387	chemo	4975	cancer	18,211	hope	142,865
cancer	12,461	node	3910	chemo	16,185	time	139,730
time	8473	cancer	3371	time	15,983	cancer	115,178
breast	8193	treatment	2366	feel	15,132	feel	109,480
treatment	6900	time	2320	dai	12,825	dai	108,850
feel	6874	posit	1996	stage	12,196	met	95,570
dai	6065	feel	1984	treatment	11,278	treatment	90,577
test	5964	dai	1885	hope	10,638	week	83,286
stage	5919	breast	1854	post	8915	pain	83,102
tumor	4833	surgeri	1808	node	8854	love	82,208
node	4683	hope	1755	breast	8775	scan	81,976
hope	4654	week	1682	week	8150	chemo	75,126
oncotyp	4642	radiat	1667	start	7600	start	73,378
week	4640	stage	1644	month	7450	month	70,849
surgeri	4472	start	1515	love	7187	hug	61,361
month	4455	tumor	1425	surgeri	6998	bone	59,894
risk	4421	test	1259	onc	6655	post	59,683
post	4348	post	1212	life	6599	onc	50,372
score	4247	month	1167	lot	6212	stage	49,436
start	4127	rad	1078	posit	6031	lot	48,270
decis	4011	lymph	999	pain	5975	new	47,497
tamoxifen	3896	mo	956	scan	5762	liver	47,182
bc	3618	scan	949	peopl	5529	hear	47,132
recurr	3598	lot	940	bc	5483	life	43,481
women	3558	told	935	live	5406	live	43,104
radiat	3508	oncotyp	918	told	5111	read	42,097
told	3504	found	864	tumor	5024	famili	41,933
onc	3275	risk	840	read	5020	peopl	41,618
posit	3267	pain	829	happi	4853	happi	38,704
read	3187	recurr	829	hug	4832	help	37,480

The results showed significant differences between the groups. Words expressing emotions and sentiments held the top position in the fourth stage forum. For example, ‘hope’ is the most frequently used word in the fourth stage forum. Additionally, words such as ‘love,’ ‘hug,’ and ‘happi’ hold high-ranking positions in the third and fourth stage forums. In the first and second stage forums, words such as ‘chemo’ and ‘treatment’ hold top positions.

## Discussion

In our online breast cancer community analysis, we found distinctive communication patterns, both in structure and content. First, the intensity of online community activation is strongly associated with disease severity. The use of the most advanced stage forum is the most active, despite its relatively small patient population compared to other patients with less advanced diseases. This is confirmed by the number of original posts, replying posts, nodes, and links. The stage 2 forum, on the other hand, was the least popular, likely due to the fact that fewer people are diagnosed with stage 2 breast cancer than with stage 1 cancer, and patients with stage 2 breast cancer report lower severity and mortality rates than patients with more advanced stage. Furthermore, the average stage dwell time of stage 2 breast cancer (1.26 years) is shorter than that of stage 1 (2.6 years) or stage 3 (4.08 years)^46^. Second, through the network structure index, we observed that the more advanced the stage, the more clusters there are in the replying network. The two types of clustering coefficients reveal more redundant links among clusters in the replying network with advancement in stages. Reciprocity also increases as the stages advance, implying that users write more replies to each other. Alternatively, the difference in the mean distance between the early and later stages is estimated to be insignificant. Finally, as cancer severity advances, emotionally-charged communication becomes dominant. More words expressing emotions appear in the replies posted in the later stages forums, as evidenced by words such as ‘hope,’ ‘love,’ and ‘hug,’ holding top positions.

These results indicated a crucial transition. As the cancer stage increases, the main purpose of the online breast cancer patient community changes from mere information exchange to emotional support. The increase in clustering coefficients was the most direct. The many closed triangles (high global clustering coefficient) and the dense connection between the nodes connected to a node (high local clustering coefficient) indicate additional and redundant connections among the nodes.^28^ If information delivery is the network’s main function, this additional connection does not make sense, as there is an existing information delivery path. That is, if A told B and B told C, A would not need to tell C again. If A has already delivered information to A’s friend, the friends do not need to tell each other the information again. However, if emotional support is the purpose, redundant connections are essential. Comfort and encouragement do not work through special information but through repeated interactions. Therefore, the rich additional connection that the high clustering coefficient indicates that the purpose of the emotional exchange is vivid in the community.

Reciprocity further confirms this finding. The high reciprocity in the later stages of forums indicates that users have emotional expectations for each other and a norm or pressure of reciprocity between them. If the purpose is to deliver information, then high reciprocity is inefficient and unnecessary. Additionally, the most frequently used words confirmed the transitions observed in the communication network structure. Medical terms related to treatment held top positions in the early stages, and emotional words held top positions in the later stages. These findings demonstrate the purpose of the replying activities. In summary, the communication patterns of online breast cancer communities are complex. Depending on the stage of breast cancer, patterns change from informational to emotional.

Our findings highlight the urgent need to consider the relational features of communication networks to expand public health policies pertaining to OHCs. Intervention through online communities is a promising public health policy. However, to maximize effectiveness, the structural characteristics of the communication networks of online communities must be considered. If public health authorities devise an online community health policy for breast cancer patients to promote proper health behaviors (e.g., screening and diet), they should develop a strategy that comprehensively considers these points.

The current study had several limitations. First, we were unable to identify the characteristics of the users, including their age, socioeconomic background, and offline network and support. According to previous studies, patients with breast cancer with better social networks and social support tend to have a lower mortality rate, a better cancer survivor prognosis, and a better quality of life.^47^ Thus, in comparison to patients with limited social networks and support, they are likely to not actively participate in OHCs; even those diagnosed with a higher stage cancer might utilize the online community for information exchange rather than for emotional support. Second, the purposes of using OHCs are known to differ between breast cancer patients and family members; patients tend to need comfort, whereas family members need information^23^. Nonetheless, we were unable to distinguish whether the individuals who posted comments and replies were patients with breast cancer or their family members or friends. Additionally, it was not feasible to determine the exact stage of breast cancer in the patients who wrote the post; one individual might leave comments and replies on different forums.

Despite these limitations, this study makes empirical, methodological, and theoretical contributions. In this study, we empirically captured the actual communication networks according to different cancer stages. Methodologically, we unveiled distinctive communication network patterns in different breast cancer forums by combining communication network and quantitative content analyses. Theoretically, we uncovered manifold functions of the OHCs. In accordance with disease severity, OHCs can serve different functions even within a particular disease condition. OHCs’ activities are more closely associated with severity than with the quantitative prevalence of stage cancer patients: the more severe the disease, the more OHC activities. Despite the relatively small percentage of breast cancer patients, the bulletin associated with the more advanced cancer stage had more active users and more intense support-seeking activities.

Practically, the distinctive communication structures show that not all users utilize online community space for the same purpose. This finding may have an important clinical implication on how to design a more effective online medical communication platform. A dual care system is strongly recommended: an information circulation format for early-stage cancer users and the emotional encouragement of interactive communication for advanced-stage users. Instead of providing a universal platform for all users, a stage-tailored support design can appease the most needed help and support. Furthermore, our results can contribute to devising interventions that transform current communication network structures to induce specific effects. Referring to Centola’s argument that networks with high clustering coefficients are advantageous for behavior propagation^14,15^, one may attempt to change the structure of the early-stage network, where clustering coefficients are low, to create a favorable environment for behavior propagation.

Future studies should consider the following points. First, although we focused on the breast cancer patient community, where most members are women, there is a possibility for future studies to examine a possible gender bias. For example, the online prostate cancer community, mostly composed of male members, may show structural characteristics different from the online breast cancer community. Second, we analyzed large-scale online data; however, studies investigating both online and offline data may be able to develop a greater understanding of why distinctive patterns emerge.

## Supplementary Information

Supplementary Information.

## Data Availability

Data collection codes can be obtained from the authors upon reasonable request. The data source is publicly available.

## Code availability

The code supporting this study is indefinitely retained for scientific and academic purposes. The code can be availed from the authors upon request.
