# Supplementary material for: Stage distinctive communication networks of the online breast cancer community
Source: Sci Rep. 2023 Jan 31;13:1726. doi: 10.1038/s41598-023-28892-7 (PMC9889398; doi:10.1038/s41598-023-28892-7)
Supplement: Supplementary file 1 — Supplementary Information. [file 41598_2023_28892_MOESM1_ESM.docx]

**Stage Distinctive Communication Networks of the Online Breast Cancer Community**

Wonkwang Jo^1,2^, Sou Hyun Jang^3^, Eun Kyong Shin^3*^

1. Department of Public Health Sciences, Graduate School of Public Health, Seoul National University
2. Institute of Health and Environment, Seoul National University
3. Department of Sociology, Korea University

* = corresponding author: Eun Kyong Shin

Contact information: Department of Sociology, Korea University, 145 Anam-ro, Seongbuk-gu, Seoul 02841 South Korea (eunshin@korea.ac.kr)

Telephone number: 82-2-3290-2089

**Supplementary Information**

Supplementary Figure S1. The distribution from simulated networks and observed value (global clustering coefficient)


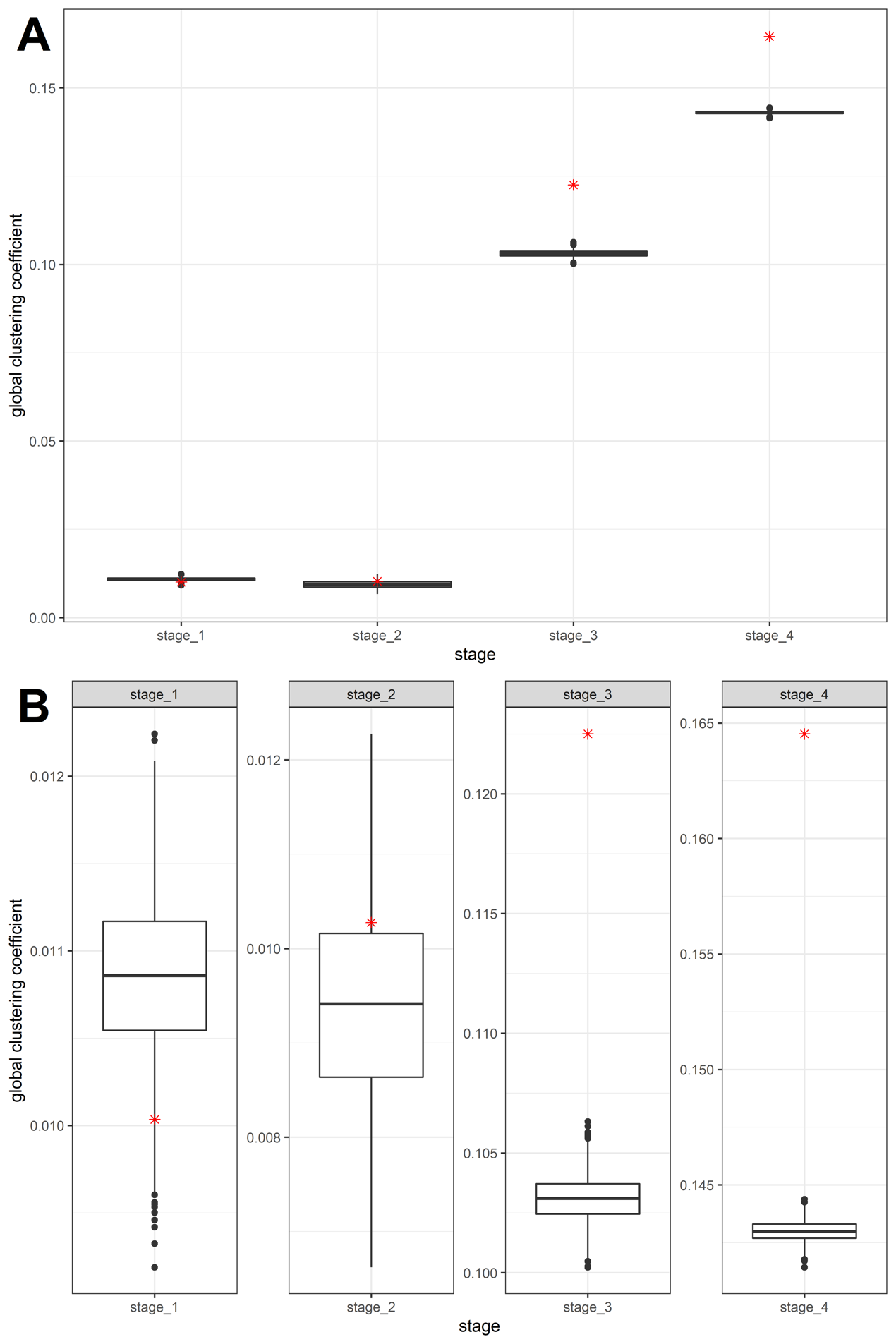


Supplementary Figure S2. The distribution from simulated networks and observed value (reciprocity)


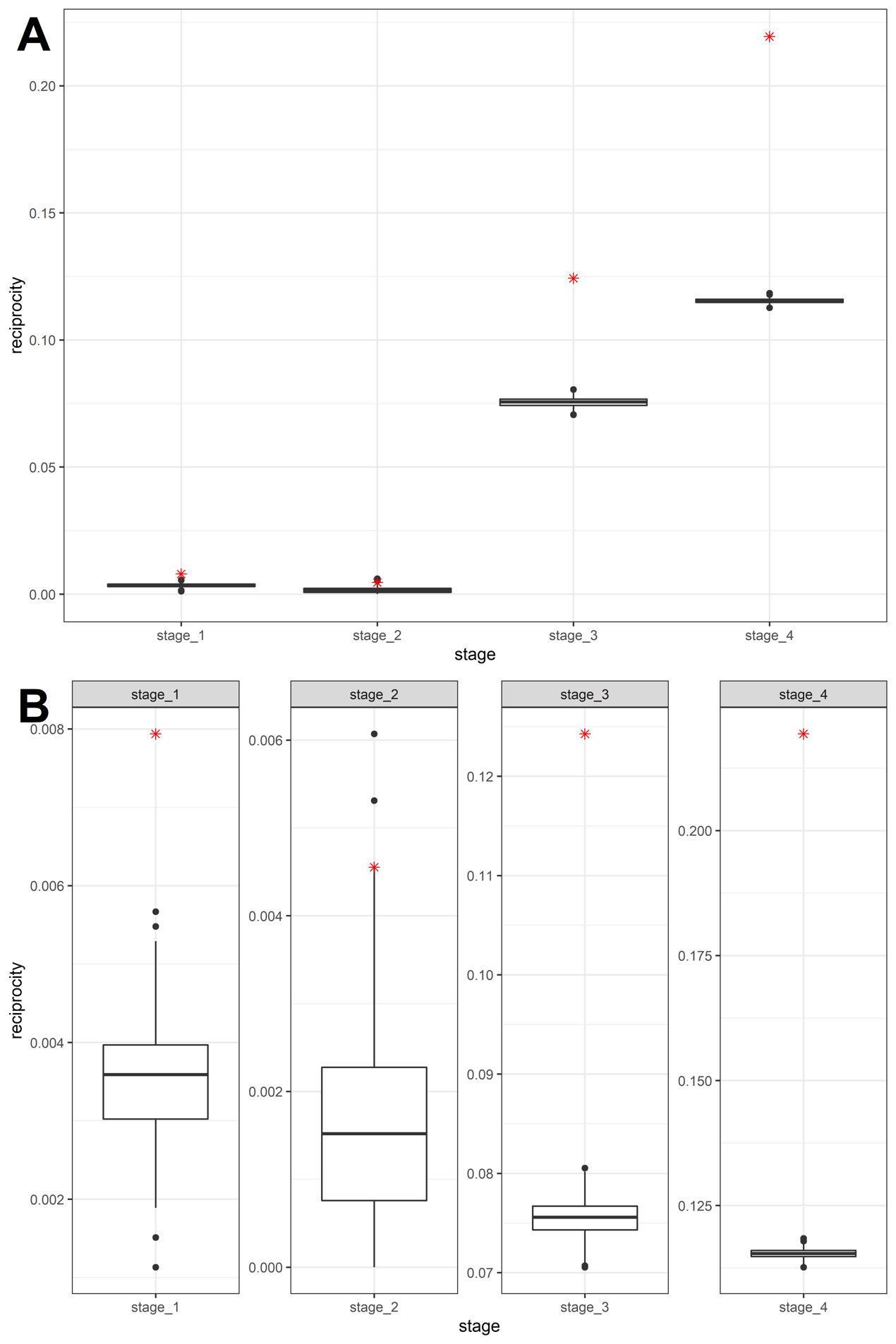


Supplementary Figure S3. The distribution from simulated networks and observed value (average local clustering coefficient)


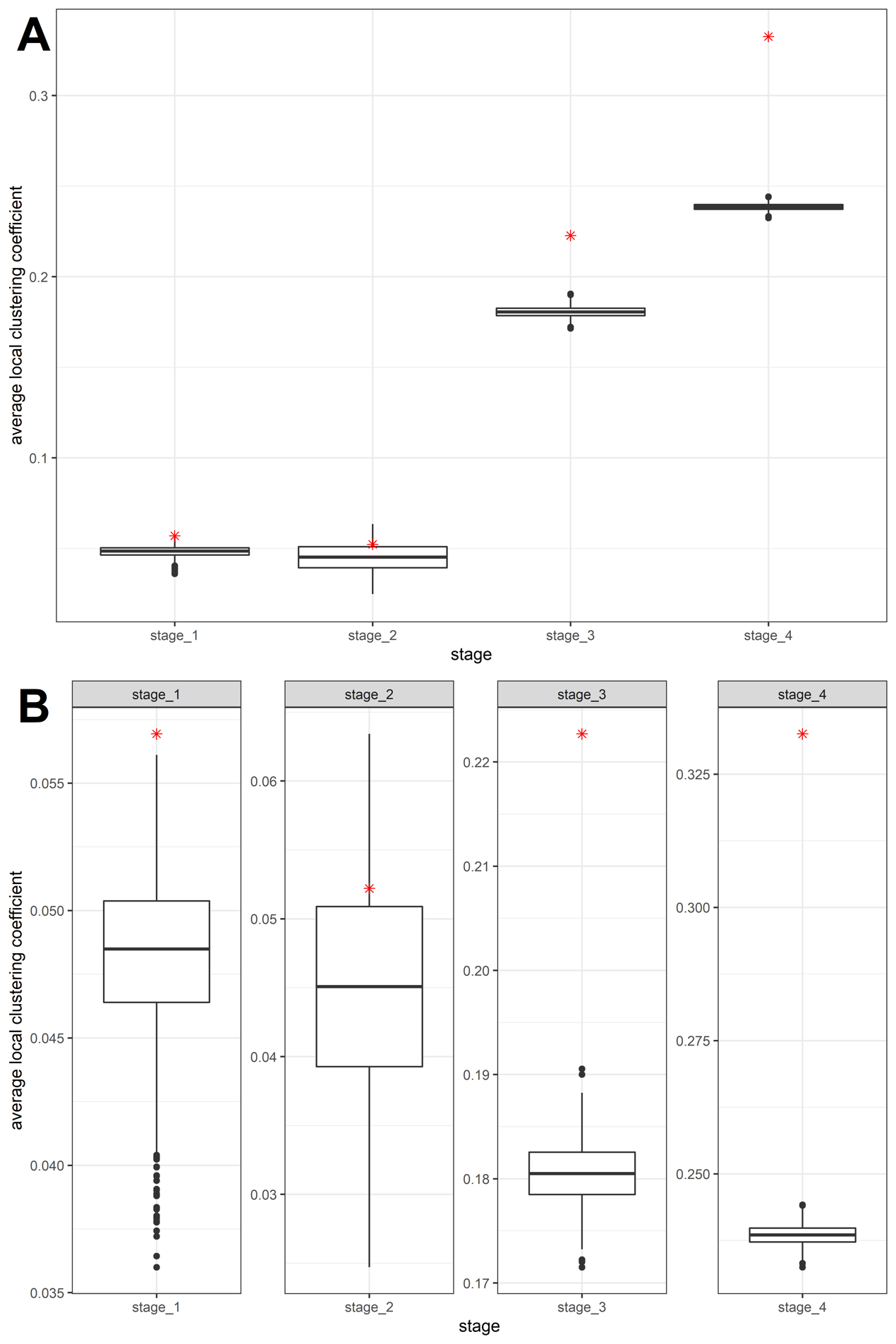


Supplementary Figure S4. The distribution from simulated networks and observed value (average distance)


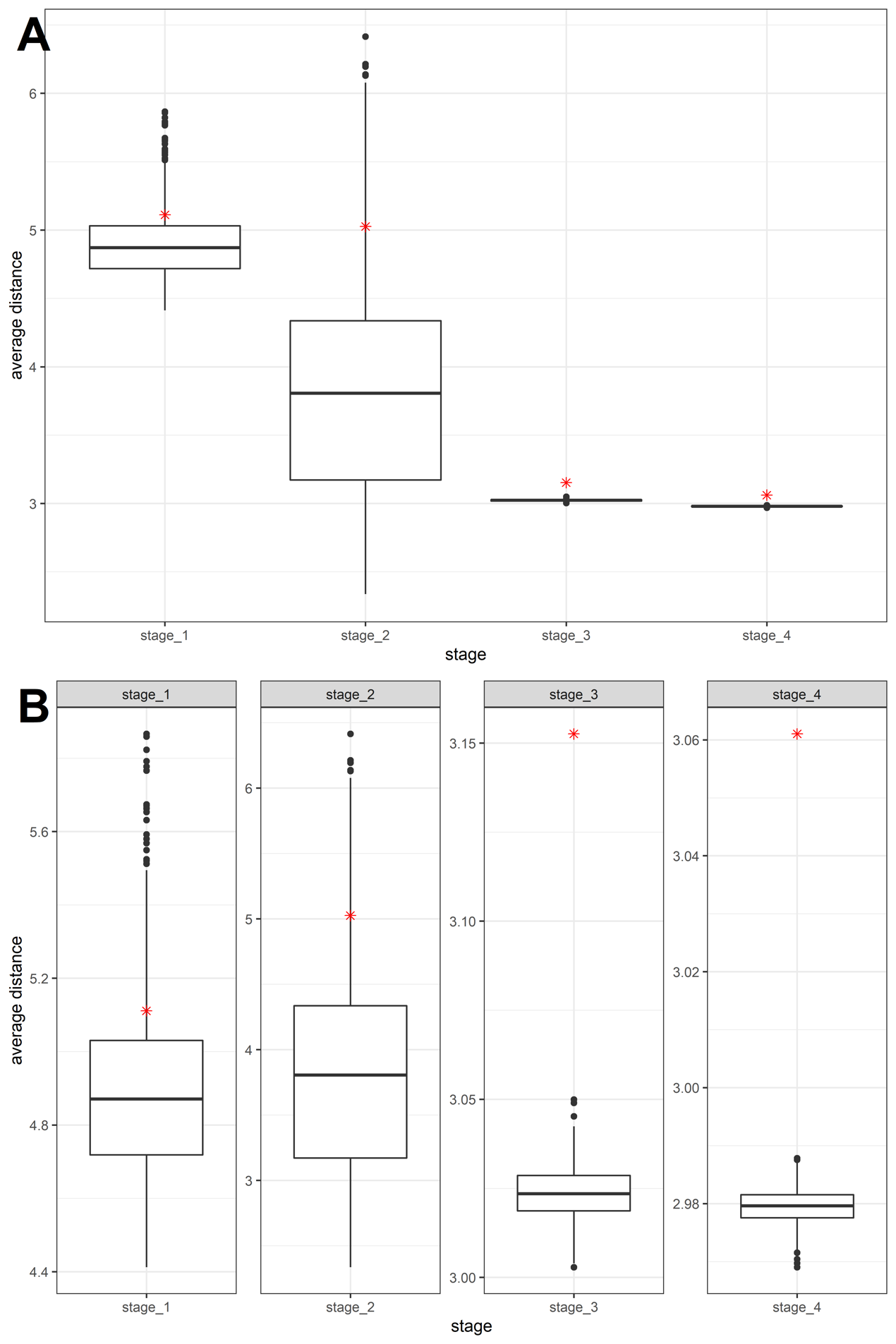


Supplementary Figure S5. The distribution of difference from simulated networks and observed difference (global clustering coefficient)


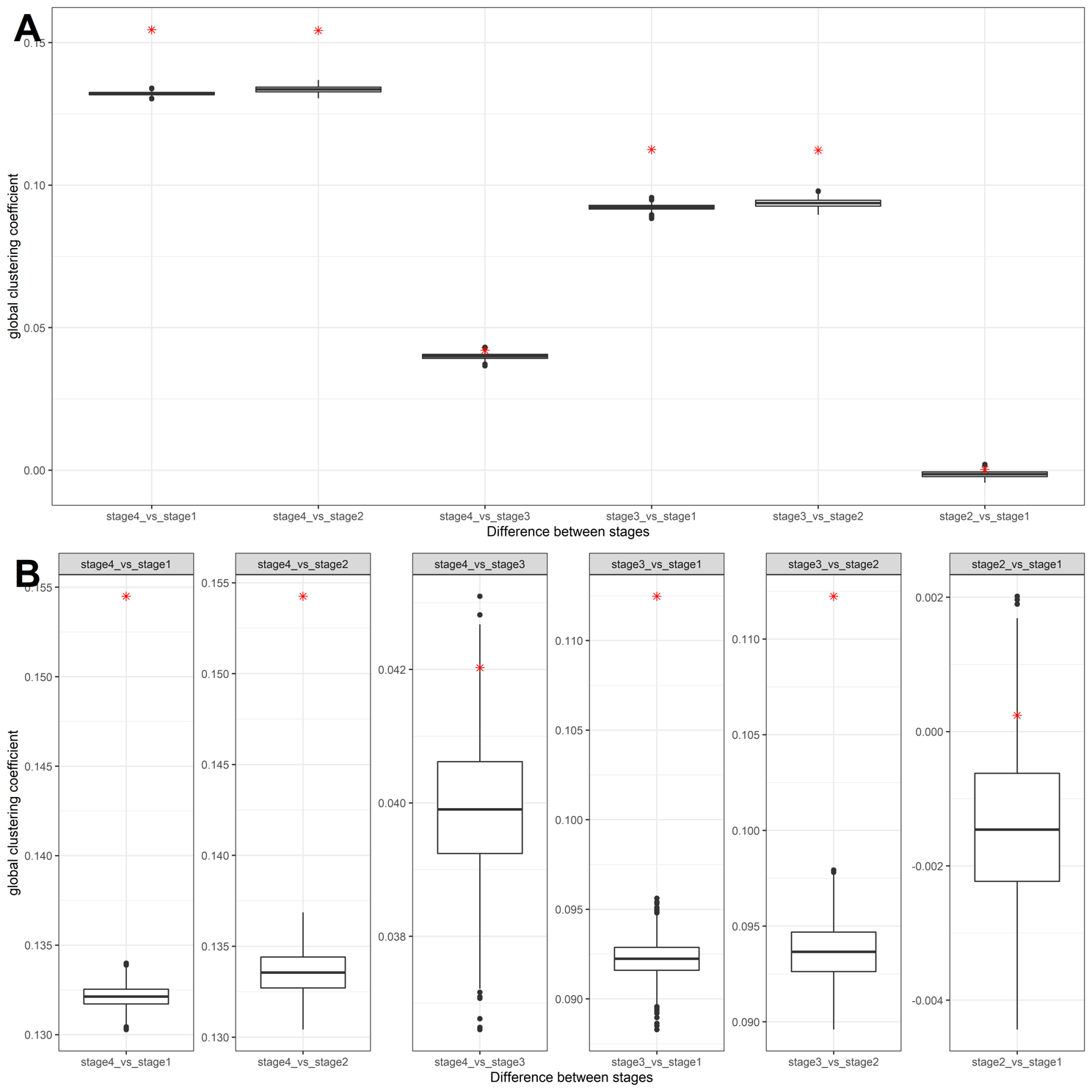


Note: “Stage A vs. Stage B” indicates the value of Stage A minus the value of Stage B

Supplementary Figure S6. The distribution of difference from simulated networks and observed difference (reciprocity)


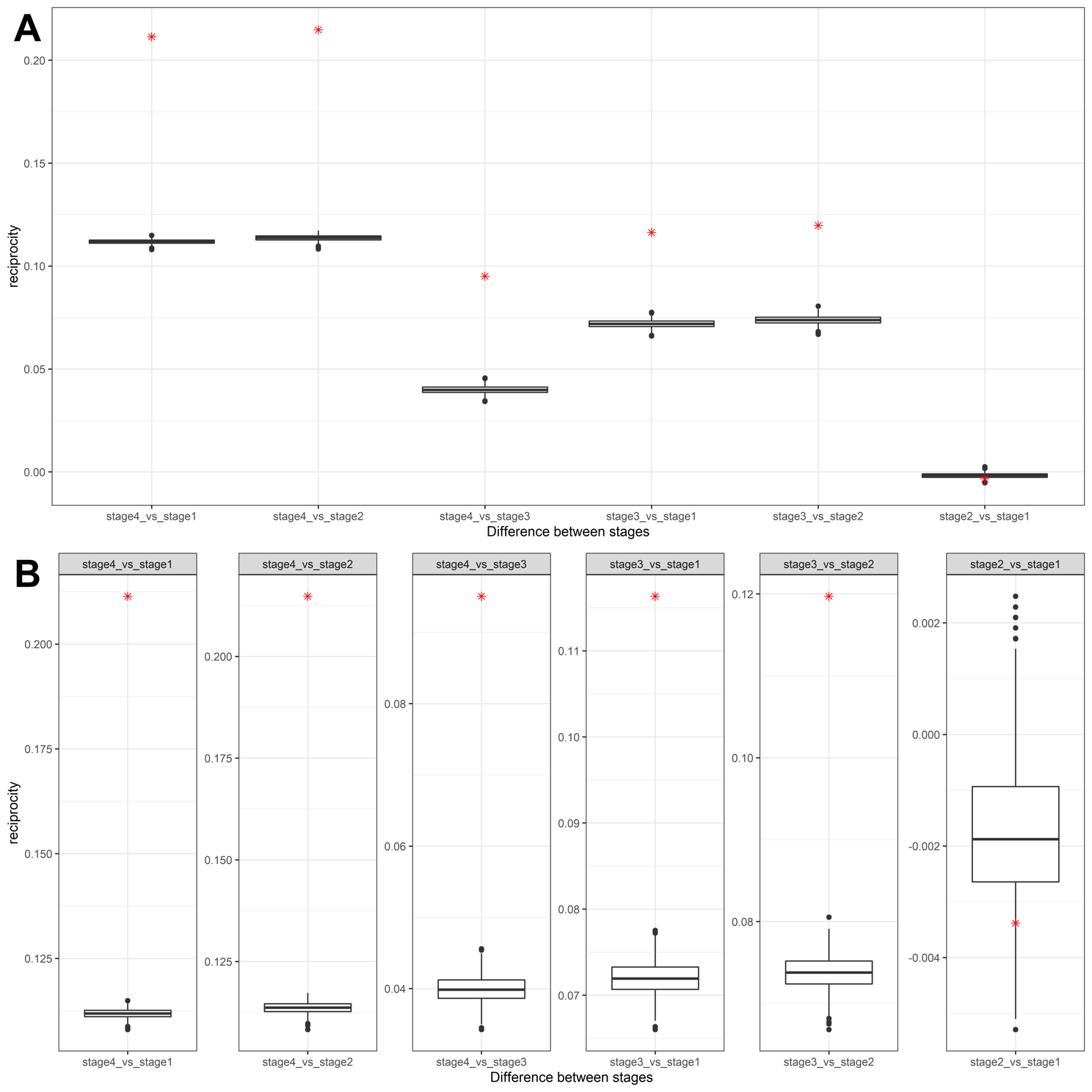


Note: “Stage A vs. Stage B” indicates the value of Stage A minus the value of Stage B

Supplementary Figure S7. The distribution of difference from simulated networks and observed difference (average local clustering coefficient)


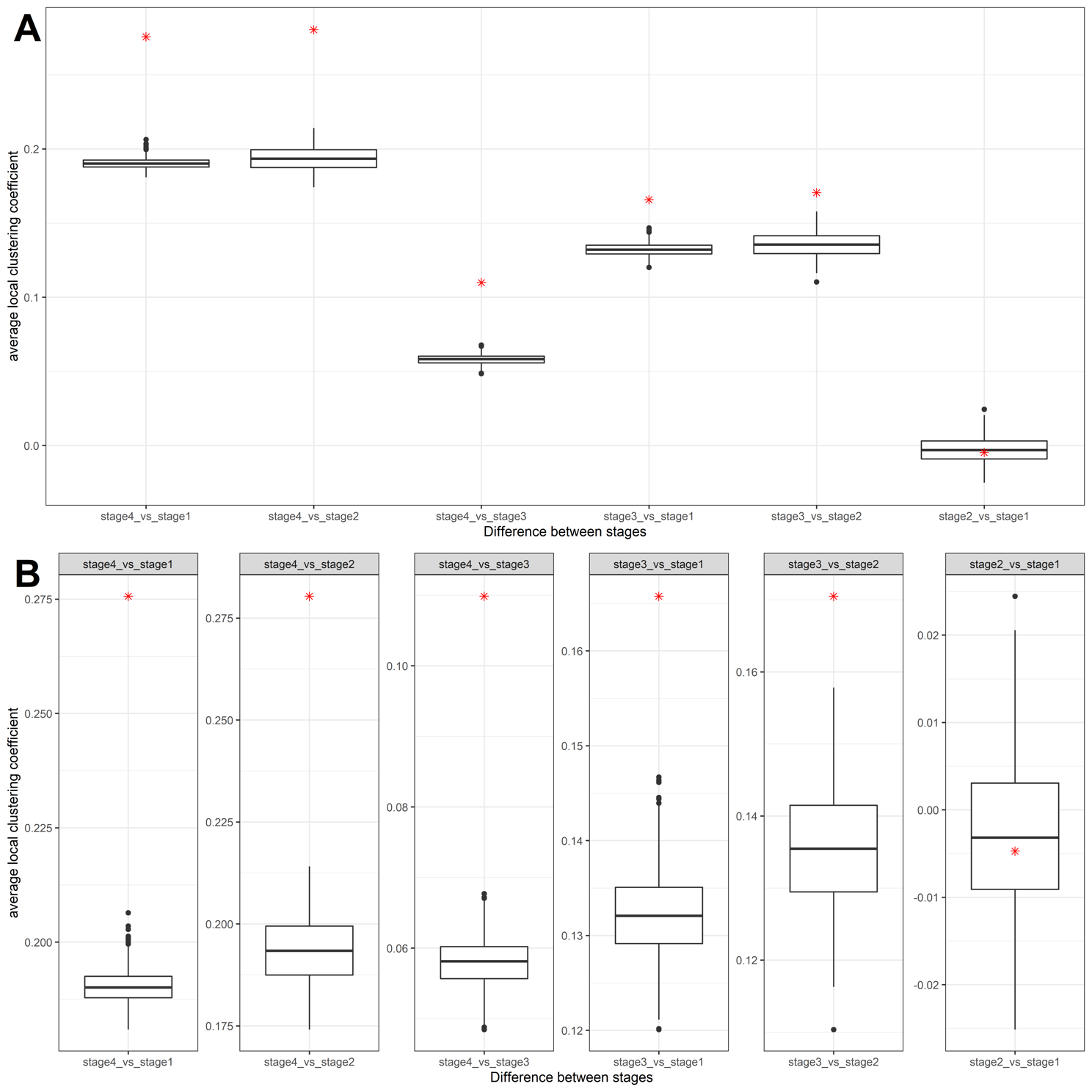


Note: “Stage A vs. Stage B” indicates the value of Stage A minus the value of Stage B

Supplementary Figure S8. The distribution of difference from simulated networks and observed difference (average distance)


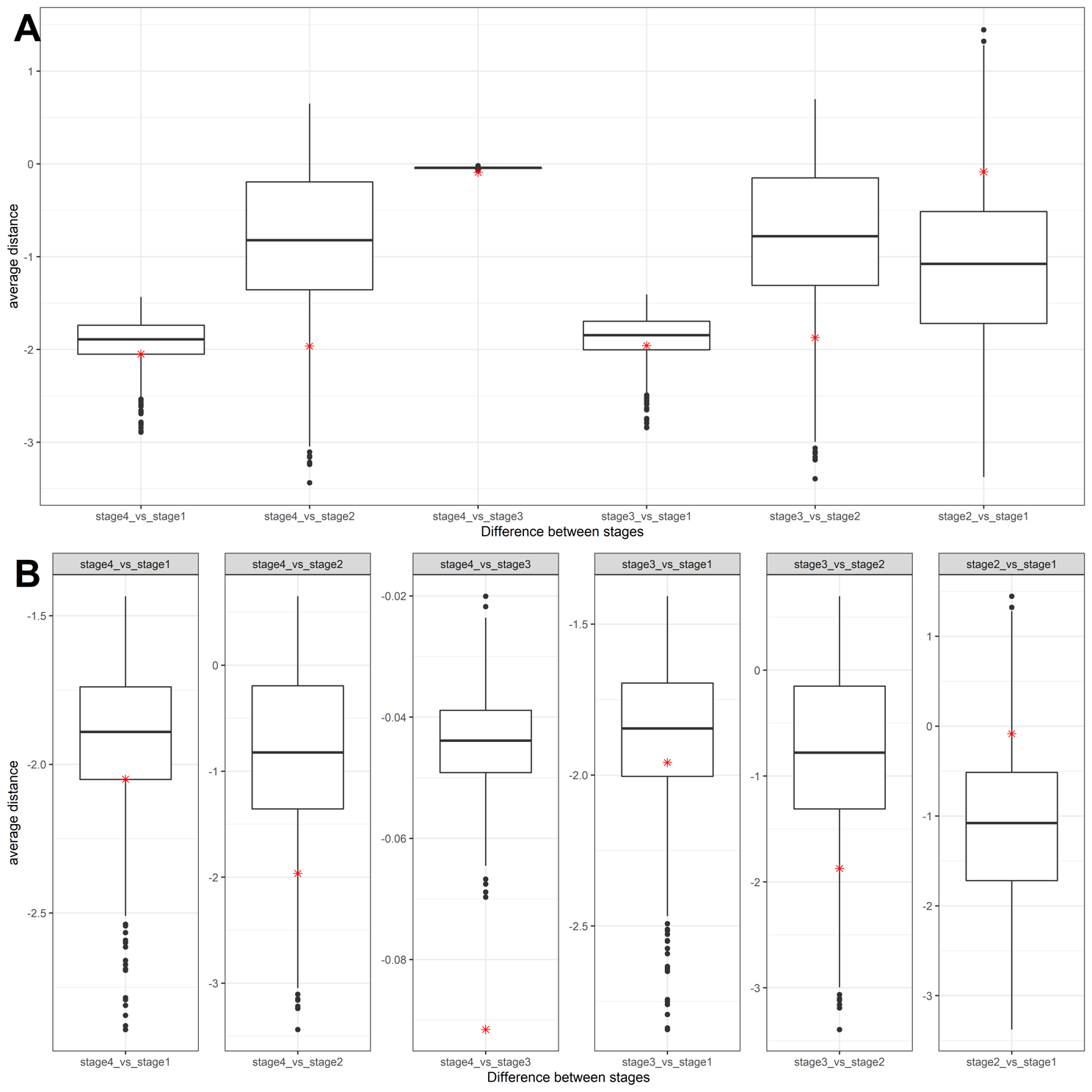


Note: “Stage A vs. Stage B” indicates the value of Stage A minus the value of Stage B
